# The Mass Spectrometric Ortho Effect for Distinguishing the Coeluting Isomers of Polychlorinated Biphenyls and the Coeluting Isomers of Polybrominated Biphenyls: Qualitative and Quantitative Aspects

**DOI:** 10.3390/molecules29153484

**Published:** 2024-07-25

**Authors:** Maurizio Masci

**Affiliations:** Council for Agricultural Research and Economics (CREA), Research Centre for Food and Nutrition, Via Ardeatina 546, 00178 Rome, Italy; maurizio.masci@crea.gov.it

**Keywords:** polychlorinated biphenyls (PCBs), polybrominated biphenyls (PBBs), mixed poly-brominated/chlorinated biphenyls (PXBs), mass spectrometry, coelution of isomers, ortho effect, analytical tool, metrology, emerging contaminants

## Abstract

Polychlorinated biphenyls (PCBs) and polybrominated biphenyls (PBBs) are persistent organic pollutants still widespread in the environment and in the food chain. Both groups of these synthetic xenobiotics consist of 209 possible congeners depending on the number and position of halogens. PCBs with the same number of chlorine atoms and PBBs with the same number of bromine atoms are isomers: ten different degrees of halogenation are allowed, which results in a lot of existing isomers for both groups. The isomers have perfect correspondence in the number and type of atoms with differences only in positioning, so their mass spectra are expected to be identical with a consequent significant analytical problem in the event of coelution of the chromatographic peaks. This is not always the case, since the mass spectrometric ortho effect is capable of effectively discriminating many coeluting PCB or PBB isomers, although not all possible ones. The present paper investigates, for the first time, the reliability of qualitative and quantitative analysis by using the ortho effect: this was conducted through targeted experimental measurements on real samples of food by using different detectors. In this context, it is shown how to recognize the presence of a PCB that does not have the ortho effect when coeluting with an isomer that has. This is an important aspect that has never been studied until now. The ortho effect is extremely simple to operate once the ordinary GC-MS runs have been performed: the analyst only needs to recheck the mass spectrum for measuring the intensity of the first dehalogenation ion. The topic is of practical relevance since two different isomers can have different health hazards, and the presence of a very toxic isomer could be masked by a less toxic one. The same mass spectrometric ortho effect also deals with PXBs (i.e., mixed poly-brominated/chlorinated biphenyls), which are emerging contaminants.

## 1. Introduction

Polychlorinated biphenyls (PCBs) and polybrominated biphenyls (PBBs) are two classes of synthetic organohalogen compounds that were largely used in the past and have now been banned (PCBs) or restricted in use (PBBs). They are still dispersed in the environment and in the food chain and present toxicity to human health, which has been well-recognized [1].

PCBs were industrially produced from the 1930s to the 1970s for the various applications such as paint and pesticide additives, dielectric oils, and many other uses [2]. Until the seventies, millions of tons entered the biosphere, and after several accidents and poisonings occurred, international bodies banned these chemicals from production and use.

PBBs have been used extensively as flame retardants in building materials, electronic devices, and furniture. In 1973, a U.S. company mistakenly shipped a flame-retardant chemical product to a livestock feed plant in Michigan instead of a nutritional supplement to be mixed in with the feed. As a result, millions of Michiganders have eaten contaminated beef, chicken, pork, milk, and eggs. The main component of the flame-retardant product was a PBB (i.e., 2,2′,4,4′,5,5′-hexabromobiphenyl, also named PBB 153) [3]. Several decades after the 1973 incident, PBBs can still be detected in the serum of adults in Michigan [4].

Although PCBs are no longer in production and PBBs are now produced to a very limited extent [3,5], exposure to these compounds is today widespread all over the world due to their environmental and biological persistence [4,6].

**Figure 1:**
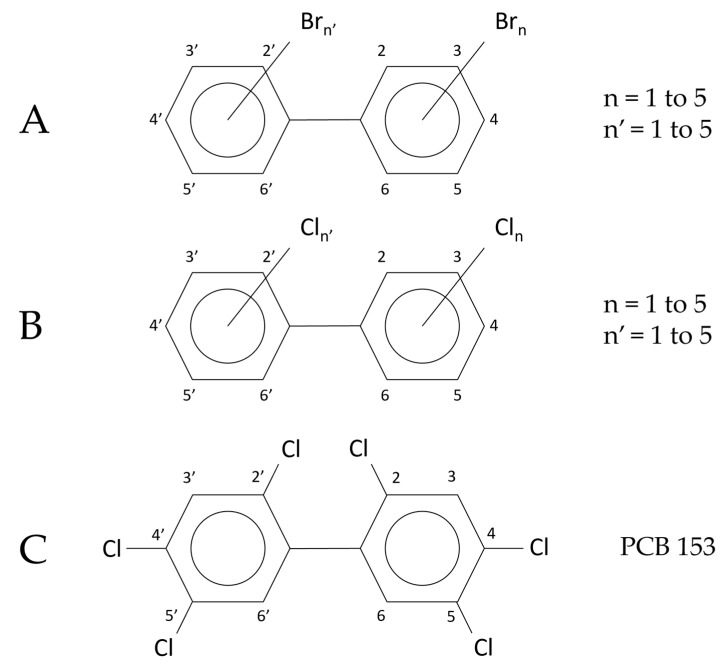
(**A**). Generic molecular structure of PBBs. (**B**). Generic molecular structure of PCBs. (**C**). Example of PCB congener: hexachlorobiphenyl, whose IUPAC name is 2,2′,4,4′,5,5′-hexachlorobiphenyl. The notation shown on the right (PCB 153) is the nomenclature introduced by Ballschmiter and Zell (numbers from 1 to 209), which is generally adopted [7,8]. A corresponding nomenclature is used for PBBs [1]. For example, 2,2′,4,4′,5,5′-hexabromobiphenyl is also referred to as PBB 153.

PCBs and PBBs have the same biphenyl ring structure, with a possible number of halogen substitutions from 1 to 10 (Figure 1).

The most used and best performing analytical technique for the analysis of PCBs and PBBs is gas chromatography, but some analytical problems are expected when two isomers chromatographically coelute with each other.

**Figure 2:**
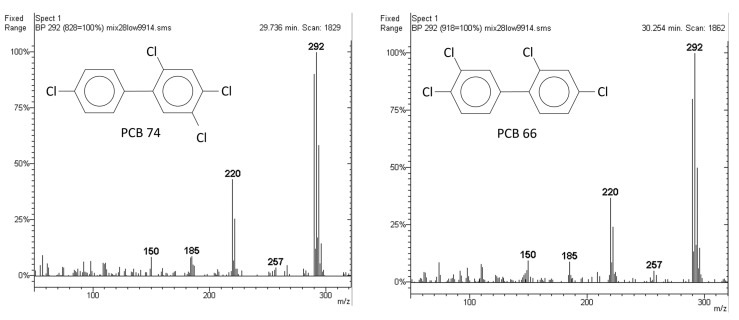
Mass spectra of PCB 74 and PCB 66, which are isomers (tetra-Cl). The mass spectra were obtained by injecting a standard mixture in the GC-MS and using the experimental conditions in Section 3.4.2. It can be noted in both spectra that the ion fragments differed from each other for the loss of one chlorine atom upon electron impact ionization (292 − 35 = 257, 257 − 37 = 220, 220 − 35 = 185, 185 − 35 = 150). The chlorine atom has a mass number of 35 or 37 (isotopic abundances: ^35^Cl about 76%, ^37^Cl about 24%). The two mass spectra were indistinguishable. In this case, the retention times were different.

In the case of PCBs, to date, there is no gas chromatographic column capable of separating all 209 PCB congeners, and the occurrence of some coelutions is a frequent phenomenon. Among them, the most probable are coelutions between the isomers (i.e., PCBs that have the same chemical formula with differences only in atom positioning); such coelutions in some cases are an unsolvable problem since the mass spectra of isomers are identical (Figure 2).

Even high-resolution mass spectrometry could not distinguish the isomers shown in Figure 2, if coalescing. In practical terms, if two isomers have the same retention time when injected as pure standards, a peak in a real sample at that retention time can be a mystery as we do not know whether it is derived from one or the other of the two isomers, or from both. This is no small matter, as there are congeners that have very high toxicity (dioxin-like) and could coalesce with non-dioxin-like isomers, so it is essential to know exactly which isomer is present in a food sample for health protection purposes. The analytical issue of PCB isomer coelution has been known for a long time and is well-present in the literature [9,10,11,12,13,14,15,16].

**Figure 3:**
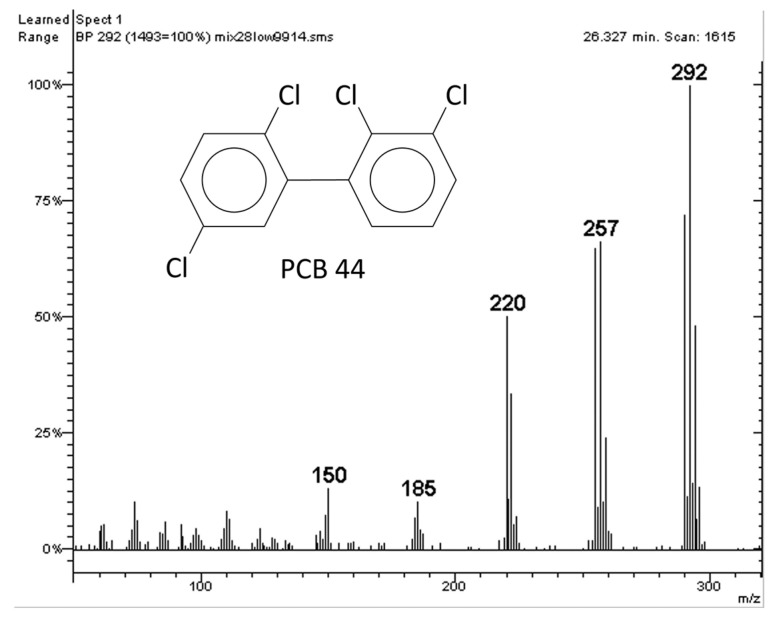
Mass spectrum of PCB 44. This was obtained by injecting a standard mixture in the GC-MS and using the experimental conditions in Section 3.4.2.

The mass spectrometric ortho effect is a phenomenon observed for 2,2′-substituted PCBs and 2,2′-substituted PBBs, which leads to an unusually high intensity of the ion deriving from the loss of one chlorine atom or one bromine atom upon ionization (first dechlorination and first debromination, respectively). In Figure 2, the first dechlorination ion is 257 *m*/*z*, where its low intensity can clearly be seen. Figure 3 shows the mass spectrum of PCB 44 as a tetra-Cl (i.e., an isomer of PCB 74 and 66 in Figure 2), but PCB 44 has a noticeable ortho effect. The high intensity of the 257 *m*/*z* ion is evident, which makes it easy to distinguish between PCB 44 and its two isomers PCB 74 and PCB 66.

The mass spectrometric ortho effect was first noticed by Oswald et al. in 1974 [17].

**Figure 4:**
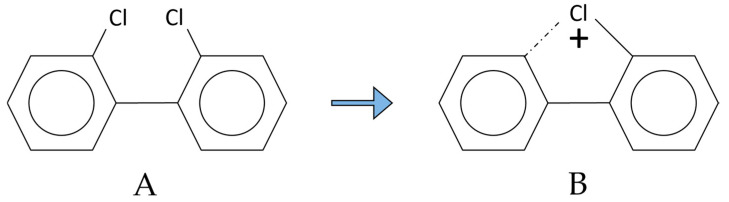
Chloromium ion intermediate (**B**) starting from a 2,2′-substituted PCB (**A**). The chloromium intermediate (first dechlorination ion) benefits from stabilization due to the formation of a 5-membered ring. The actual process involved in the rate of formation of the first dechlorination ion may involve the displacement of one ortho chlorine by an ortho chlorine from the other ring of the biphenyl nucleus. Data from the ion kinetic energy spectra support this pathway [18].

Levy and Oswald in 1976 [18] hypothesized that the intense formation of the first dechlorination ion could occur via a 5-membered chloronium ion intermediate (Figure 4), a species that has been suggested to be important in the fragmentation of aliphatic chloro-compounds.

Sovocool and others in 1987 extended the study of the ortho effect to PBBs [19].

In 2013, Osemwengie and Sovocool studied the ortho effect for all 209 PCB congeners. With the gas chromatographic column of high performance that they used 60 PCBs coeluted with an isomer. Of that number, 19 PCBs could be clearly distinguished via the ortho effect. They highlighted how the ortho effect is important in recognizing the isomers of planar PCBs because they have a special toxicity profile as they are dioxin-like [20].

Apart from these studies, there has been no other research on the ortho effect and apart from one isolated case [21], there have been no practical applications to real samples.

In the present work, the qualitative and quantitative analytical reliability of the ortho effect for PCBs were verified on real samples. This was conducted by using gas chromatography coupled to mass spectrometry and, for the first time, with other detectors, to compare the independent techniques. An application of the ortho effect to PCBs, published in a recent paper, is discussed.

An overview of the ortho effect related to polybrominated biphenyls is also reported. This works in a completely similar way to that observable for PCBs: the fact that planar PBBs have the same dioxin toxicity profile as planar PCBs is included in the discussion, hence the analytical importance of the ortho effect for PBBs.

Finally, a mention is made of the ortho effect relating to PXBs (mixed poly-brominated/chlorinated biphenyls) to be considered as a class of emerging contaminants because they have only been known for a short time. The possible combinations give rise to 9179 potentially existing PXBs, with the toxicological profiles of many of these of considerable interest (planar PXBs) and with considerable analytical problems, for which an additional analytical tool such as the ortho effect could potentially be very useful.

## 2. Results and Discussion

### 2.1. Polychlorinated Biphenyls (PCBs)

**Figure 5:**
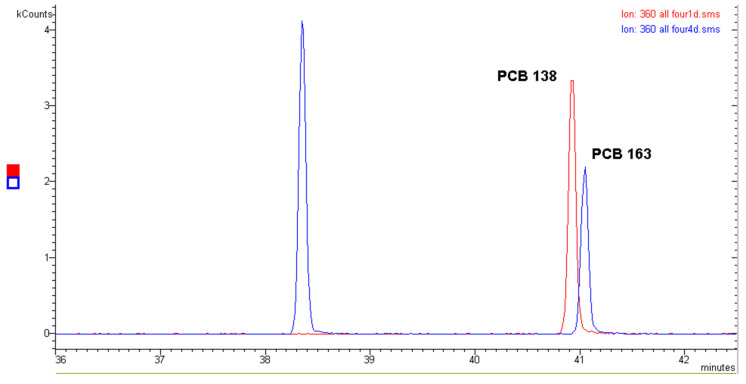
GC-MS overlaid chromatograms of standard PCB 138 (200 µg L^−1^) and PCB 163 (100 µg L^−1^) injected separately. Chromatograms were obtained by displaying the molecular ion 360 *m*/*z*. Experimental conditions are the same as in Section 3.4.2.

**Figure 6:**
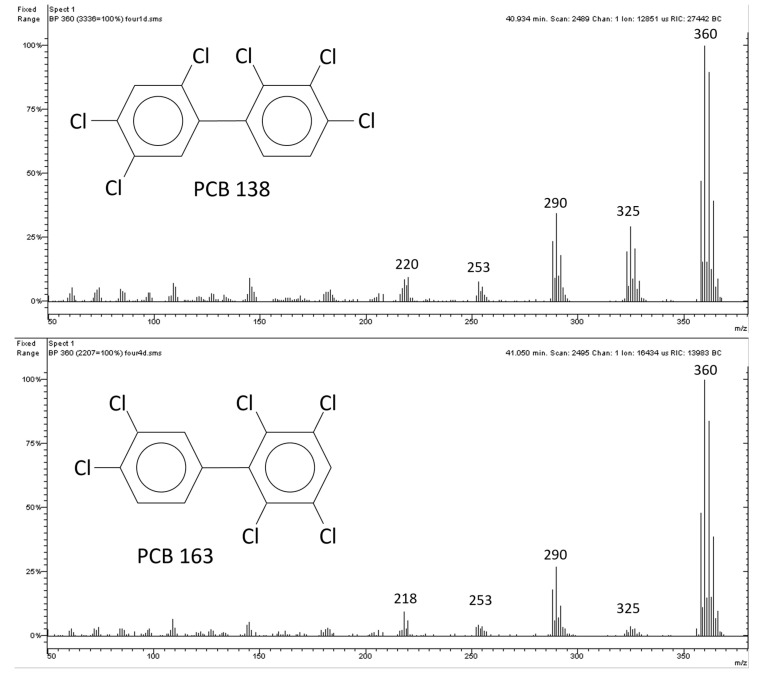
Mass spectra of PCB 138 and PCB 163 relating to the peaks in Figure 5.

A frequent PCB isomer coelution is the one between PCB 163 and PCB 138 [20]. Among other things, PCB 138 is listed in European Union Regulation No. 915/2023 as one of the six indicator PCBs to be determined in foodstuffs [22]. In the present work, the pure standards of PCB 138 and 163 were injected preliminarily to control the retention times on the column used and the mass spectra. Results are shown in Figure 5 and Figure 6.

As expected, the 2,2′-substituted PCB 138 had an intense first dichlorination ion (325 *m*/*z*) compared to PCB 163, which is to say that PCB 138 has a significant ortho effect and PCB 163 does not. The precise measurement of the ortho effect is shown below. The first dechlorination ion is usually indicated by the notation [M-Cl]^+^, while the molecular ion is indicated by the notation [M^●+^]. The ortho effect is calculated according to Equation (1):

(1) $$\frac{{\left[M-Cl\right]}^{+}}{\left[M^{•+}\right]}\times 100$$

This represents the percentage intensity of [M-Cl]^+^ (the most intense ion in the dechlorination cluster) compared to [M^●+^] (the most intense ion in the molecular cluster). In Figure 6, the molecular ion [M^●+^] of PCB 138 is 360 *m*/*z*, for which it was measured an intensity of 3336 counts, while for the first dichlorination ion [M-Cl]^+^ = 325 *m*/*z*, it was measured an intensity of 979 counts. Therefore, the ortho effect for PCB 138 in this mass chromatographic run was 29%:

(2) $$\frac{979}{3336}\times 100=29$$

The same calculation for PCB 163 in Figure 6 yielded 4%. The ortho effect is considered ‘substantial’ when it is ≥17% [20]. In Table S1 (Supplementary Materials), the intensities of the ortho effect measured in the experimental conditions of the present work are reported for 37 PCBs. The values obtained were substantially in agreement with previous works [20], and it was possible to deduce that the ortho effect had significant intensity in all PCBs with chlorine atoms in the 2,2′-substituted positions. Only when a 6,6′-substitution was contemporarily present did the ortho effect not occur.

**Figure 7:**
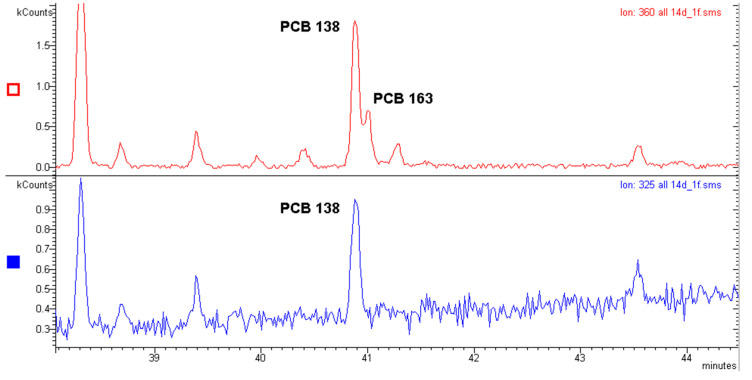
GC-MS chromatogram of Food Sample 1. (**Top**) Chromatogram obtained by displaying the molecular ion 360 *m*/*z*. (**Bottom**) Chromatogram obtained by displaying the ion 325 *m*/*z*. Sample injected using the same experimental conditions as in Section 3.4.2.

Under the same conditions shown in Figure 5 and Figure 6, a real sample of food was analyzed (Figure 7).

Figure 7 shows that both PCB 138 and 163 were present in the food sample where they almost coeluted.

It can be observed that the quantitative analysis carried out with the 325 *m*/*z* ion of the first dechlorination allowed for the determination of PCB 138 in Food Sample 1 without any interference of PCB 163 as the 325 *m*/*z* ion of the latter did not emerge at all from the baseline: this is a common observation for PCBs with a negligible ortho effect and injected at concentrations below 60–70 µg L^−1^ (Figure S1, Supplementary Materials).

By carrying out the analysis of Food Sample 1 as detailed in Section 3 and by using the 325 *m*/*z* ion of a PCB 138 standard solution for calibration purposes, a concentration of 26.9 ng g^−1^ for PCB 138 in Food Sample 1 was obtained.

It must be noted that the sensitivity was lower when the ortho effect was used in place of the molecular ion. This is due to the lower intensity of the first dechlorination ion with respect to the molecular one. For PBBs, the opposite is true, as we will see later. In the case of PCB 138 in Figure 7, the signal to noise ratio (s/n) was 45 for the molecular ion 360 *m*/*z* and 8 for the 325 *m*/*z* ion. This implies a LOQ of 1.8 ng g^−1^ when the 360 *m*/*z* ion is used and a LOQ of 10.0 ng g^−1^ when the 325 *m*/*z* ion is used. This is by considering the value of s/n = 3 as a necessary criterion for the quantification. It can be noted that PCB 138 in Figure 7 had an ortho effect of 40%, causing a value of s/n equal to 8 for the 325 *m*/*z* ion. It can be deduced that an ortho effect of 17% would cause an s/n = 3 for the same ion. The value 17% thus represents a threshold value below which the quantification of PCB 138 via the ortho effect would not be possible in the present case.

An important verification is the reliability of the quantitative analysis using the ortho effect. To achieve this goal, nine PCBs were quantified in Food Sample 1 in Figure 7 by using and comparing with each other the quantitative analyses carried out with three different techniques:

(a) The quantitative analysis performed with the molecular ion [M^●+^];
(b) The quantitative analysis performed with the first dechlorination ion [M-Cl]^+^ (ortho effect);
(c) The quantitative analysis performed with an electron capture detector (GC-ECD).

Only PCBs with a substantial ortho effect and without any coelution were included in the cross-check, for example, from this verification, PCB 138 was excluded because the coelution with PCB 163 would hinder proper quantification by using both the molecular ion and the ECD detector. As Table 1 shows, the quantitative analysis carried out with the [M-Cl]^+^ ion showed good reliability, since agreement with the analysis performed with the molecular ion and with the ECD detector was satisfactory. The deviation between the quantification with [M-Cl]^+^ and the quantification with [M^●+^] was 11% on average, while the deviation between the quantification with [M-Cl]^+^ and the ECD detector was 13% on average. The deviation between the quantification with [M^●+^] and the ECD detector was 8% on average. These values fall well within the uncertainty range specific to this type of trace analyses [23].

Table 2 reports the LOD and LOQ of the different detection methods. Given the aspect of chromatograms and the good shape of the peaks (both with the ECD detector and mass spectrometry), a peak with a signal to noise ratio of 2 was deemed sufficient for the LOD evaluation while for the LOQ evaluation, a signal to noise ratio of 3 was considered as necessary. The following sensitivity scale can be observed: ECD > GC-MS, [M^●+^] > GC-MS, [M-Cl]^+^. With regard to specificity, the ECD detector was not at the reliability level of mass spectrometry. A complete comparison between MS and ECD detectors in analyzing PCBs has been reported in our previous work [24].

**Figure 8:**
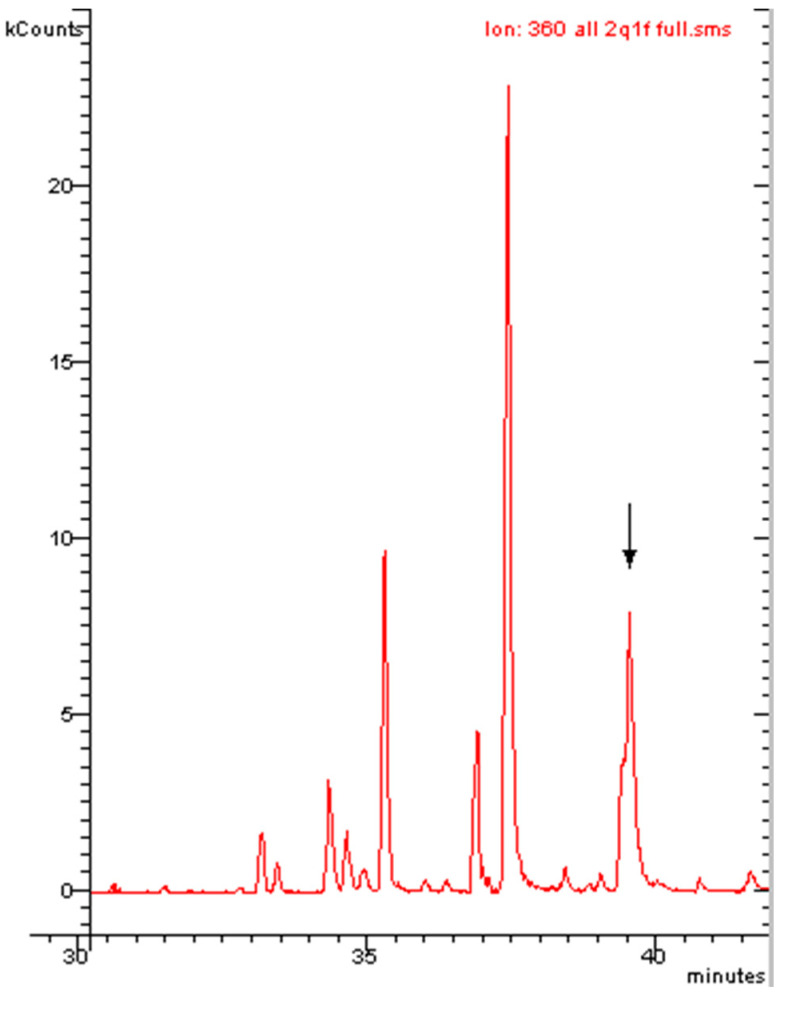
GC-MS chromatogram of Food Sample 2. Indicated by the arrow: PCB 138 + 163. Experimental conditions the same as in Section 3.4.3.

The observation in Table 1 that the quantitative analysis of single peaks produces the same result whether conducted with [M^●+^] or [M-Cl]^+^ is also important for the isomers with no ortho effect. Indeed, in the presence of a coelution between an isomer that does not have the ortho effect and an isomer that does, the quantitative analysis with the molecular ion [M^●+^] will be very different from that carried out with the first dechlorination ion [M-Cl]^+^. This is because the PCB without the ortho effect will contribute to the [M^●+^] signal but not to the [M-Cl]^+^ signal. Therefore, every time the quantitative analysis with [M^●+^] gives a much greater result than the quantitative analysis with [M-Cl]^+^, a PCB without the ortho effect is most likely to be present in the studied peak. To demonstrate this last assertion, a real sample of food was injected under different experimental conditions and with a different column (Figure 8).

**Figure 9:**
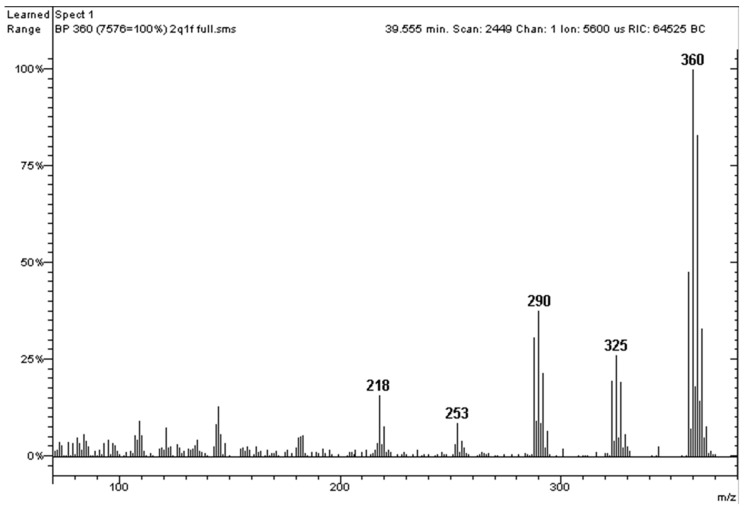
Mass spectrum of the peak indicated by the arrow in Figure 8.

With the conditions in Figure 8, PCB 138 (with ortho effect) and PCB 163 (with no substantial ortho effect) practically coeluted. The mass spectrum of the peak identified it as a hexachlorobiphenyl (Figure 9). The quantitative analysis of this hexachlorobiphenyl provided the value 101.3 ng g^−1^ when conducted with the molecular ion 360 *m*/*z* and a value of 69.3 ng g^−1^ when conducted with the first dechlorination ion 325 *m*/*z*. The deviation of 68% confirms the presence of a coelution, and the aspect of the mass spectrum, typical of hexachlorobiphenyls, ensured that we were dealing with an ortho effect–non ortho effect coelution. Therefore, in the case of isomer coelution in real samples, this could represent a useful method for discovering the presence of PCBs that do not have the ortho effect. Among the latter, there are very toxic congeners called ‘dioxin-like’.

The ortho effect technique is applicable in a variable number of cases, depending on the experimental conditions used. In the conditions of Osemwengie and Sovocool [20], who injected all 209 PCBs on a column of high separation power, 60 PCBs coeluted with an isomer, and in 19 cases, the ortho effect was decisive in solving the problem. Using a less specific column would increase this number, as detailed in Section 2.2.

Possible coelutions of peaks from complex matrices having the same ion fragment and the same retention time of the first dehalogenation ion are readily solvable by using high resolution mass spectrometry. In fact, the interferent from the matrix will be a different compound, with a different chemical formula and a consequent different exact mass.

#### Recent Application to a Real Case

In 2023, a relevant analytical problem was solved by the application of the ortho effect [23]. During the analysis of organochlorines in shortenings, the authors observed an unexpected peak related to a tetrachlorobiphenyl. The authors knew that with the column used, three different isomers of tetrachlorobiphenyls could coelute at that retention time: PCB 47, PCB 62, and PCB 65. The only way to solve the problem was to check for the possible presence of the ortho effect, since among PCB 47, 62, and 65, only PCB 47 has a substantial ortho effect. Indeed, the peak in the shortening samples had an intense ortho effect. The problem was solved, which allowed the authors to conduct a targeted literature search until they discovered that PCB 47 is currently unintentionally released into the environment by silicone rubber factories [25,26].

### 2.2. Polybrominated Biphenyls (PBBs)

PBBs are the brominated analogs of PCBs. When compared to PCBs, the scale of diffusion of PBBs into the environment via the atmosphere is lower for various reasons such as the larger size and significant photo lability [27]. Their toxicity is well-established since the planar configured PBBs have a common mechanism of toxic action to chlorinated dioxins and dioxin-like PCBs via binding to the aryl hydrocarbon receptor (AhR). Many studies have shown that some planar PBB congeners are among the most active inducers of AhR in aquatic food chains, foods, and humans and contribute, albeit at a lower level, to the cumulative burden of dioxin-like toxicity [27,28,29,30,31]. On the other hand, among non-planar PBBs, PBB 138, which accounts for 5–12% of the widespread commercial mixture FireMaster, has raised suspicion of anti-androgenic activity [32]. This highlights the importance of analytically distinguishing between the various isomers of polybrominated biphenyls as one isomer can have a completely different degree of toxicity from another.

In Table S2 (Supplementary Materials), the mass spectra and intensity of the ortho effect are reported for 17 PBBs, based on the NIST 08 MS library. It was observed that the ortho effect manifests itself in a similar way to PCBs, but with some differences. The rule of the presence of the ortho effect with the 2,2′- substitution was confirmed, but the intensity of the first debromination ion took on more extreme values. When present, the average value of the ortho effect was 159%, with values above 140% in seven out of eight cases. This suggests a better sensitivity of the ortho effect for PBBs compared to the use for PCBs: if the quantitative analysis is performed with the first debromination ion, the sensitivity will be better compared to the molecular ion. A separate case can be mentioned for the 2,2′,6,6′-substitution, where the lack of ortho effect, already seen for PCBs, was confirmed, but with an intensity of the first debromination ion that was slightly higher than that measured for 2-, 2,6-, or no-ortho-substitutions (which have an average value of 0.4%).

The practical application of the ortho effect to polybrominated biphenyls was foreseen in a 2021 work by Wang et al. investigating PBBs in serum from people living in an electronic waste dismantling area in China [3]. In their study, they analyzed 35 PBB congeners and predicted the GC retention times of other 171 congeners by applying linear regression models. The authors used a 30 m capillary column of low polarity (5% diphenyl 95% dimethyl polysiloxane) for general MS use. By examining the GC retention times for 206 PBBs as predicted by Wang et al., it can be concluded that with their chromatographic conditions, 122 PBBs would have coalescence problems with an isomer, but 28 of them would be distinguishable thanks to the ortho effect.

### 2.3. Mixed Poly-Brominated/Chlorinated Biphenyls (PXBs)

**Figure 10:**
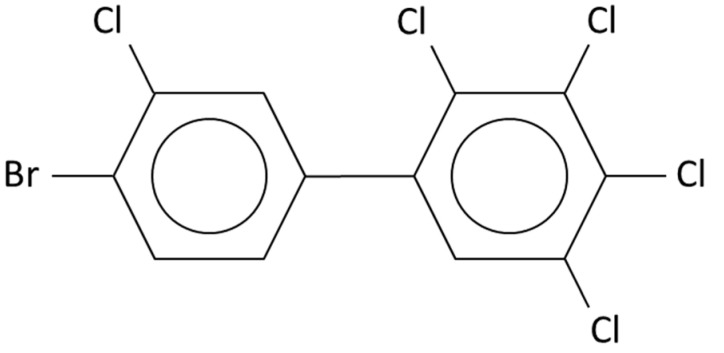
4′-Br-2,3,3′,4,5-Cl-biphenyl. This molecule was investigated in a 2011 research study by Fernandes et al. [33]: it is an analog to PCB 156 and PBB 156. Depending on the position of the bromine atom, there are six possible PXB 156. Due to the absence of 2,2′-substitution, it is to be expected that the ortho effect is absent for the six possible PXB 156.

Mixed poly-brominated/chlorinated biphenyls (PXBs) are a new class of emerging contaminants similar to PCBs and PBBs, which have both bromine and chlorine atoms as substituents on the biphenyl ring (Figure 10).

These xenobiotics were never intentionally produced but are formed as by-products during the combustion process of plastics and electrical equipment containing materials such as polyvinyl chloride and brominated flame retardants. All combinations of Cl and Br atoms on the biphenyl ring make the theoretical existence of 9179 congeners of PXBs possible. Their presence in the environment, food, humans, animals, and milk has been detected for a few years all over the world, so they should therefore be considered as emerging contaminants. Of particular attention are the dioxin-like congeners of PXBs, on which toxicity studies are being conducted that confirm their danger to human health, being even higher than dioxin-like PCBs. Among the dioxin-like PXBs, there are 62 congeners of the non-ortho-substituted type plus many others of the mono-ortho-substituted type [33,34,35,36,37,38,39,40].

For PXBs, there is a limited availability of pure standards and the availability of PXB mass spectra in most mass spectra databases is very limited, if not existent. This may create difficulties in both the recognition of a single congener in a real sample and in the possibility of studying the ortho effect in detail. Nevertheless, some hypotheses are possible. PXBs with a 2,2′-Cl- or 2,2′-Br-substitution almost certainly have the ortho effect excluding the case of 2,2′,6,6′-substitution. This is also the case of the converse: the absence of a 2,2′-substitution almost certainly coincides with the absence of the ortho effect. It is reasonable to suppose that the very high number of possible PXB congeners leads to many chromatographic coelutions with an associated large number of coelutions between isomers. The latter cannot be resolved, even with high resolution mass spectrometry. Therefore, an analytical tool like the ortho effect that allows for some simplification can only be welcome. For example, all planar dioxin-like PXBs do not have the ortho effect, which is a first important simplification.

## 3. Materials and Methods

Measurements conducted in this study were carried out as previously published [21,23,24,41] and according to the United Nations ‘Guidance on the global monitoring plan for POPs’ [42]. The instrumentation used was as required by the cited document (page 86, Table 5.1, levels 2a and 3). The experimental procedure is briefly summarized below.

### 3.1. Reagents

All reagents used were of pesticide grade. Acetonitrile, acetone, isooctane, n-hexane, petroleum ether 40–60 °C, methyl alcohol, dichloromethane, sodium sulfate, and Florisil^®^ 60–100 mesh were purchased from Carlo Erba Reagents (Milan, Italy). The Supelclean LC-18 solid phase was from Supelco/Sigma-Aldrich (Bellefonte, PA, USA). Pure standards of PCBs were purchased from two different producers, mainly in solution form: certified solutions, as single standards or mixtures, were from Dr. Ehrenstorfer (Augsburg, Germany) and from AccuStandard Inc. (New Haven, CT, USA).

### 3.2. Food Samples

Food samples analyzed in the present study were fish fillets purchased from local markets (Food Sample 1: *Silurus glanis*) or from the practice of sea cage aquaculture (Food Sample 2: *Thunnus thynnus*). These samples were chosen on the basis that they generally have a significant concentration of PCBs.

### 3.3. Sample Preparation

Sample preparation was conducted as previously reported [41]. Briefly, the fish fillets were homogenized, solvent-extracted, and subjected to purification steps with the solid phase extraction technique. In the first purification, diatomaceous earth and C18 were used as the stationary phases; elution was carried out with acetonitrile. In the second purification, the solid phase was constituted by Florisil^®^ while elution took place with n-hexane. To monitor each analysis, three internal standards (surrogates) were exploited; these were added to each sample immediately after the extraction step: a 100 μL solution containing PCB 5 at 350 μg L^−1^, PCB 198 at 500 μg L^−1^, and 2,2′-DDE at 304 μg L^−1^ was used.

### 3.4. Instrumental

The instrument used was a Varian^®^ 3800 GC (Palo Alto, CA, USA) with the possibility of housing two capillary columns, the first connected to an electron capture detector (ECD) and the second to the mass spectrometer (Ion Trap Saturn 2000^®^, Palo Alto, CA, USA).

#### 3.4.1. GC-ECD Analysis

The capillary column, RTx^®^-PCB Restek (Bellefonte, PA, USA), 60 m × 0.25 mm ID, 0.25 µm df, was connected to the ECD. Injections (1 µL) were performed in splitless mode: split off at 0.0 min, split ratio 100 at 0.75 min, split ratio 20 at 3 min. The injector temperature was 280 °C. The column oven temperature was programmed as follows: initial temperature 100 °C, 2.67 min hold; raising to 200 °C at a rate of 30 °C/min, 0 min hold; raising to 320 °C at a rate of 2 °C/min, 1 min hold. Helium, as the carrier gas, was used at a constant flow of 1.1 mL/min. The ECD temperature was 300 °C. According to the UNEP guidelines [42], the addition of a recovery standard to check the solvent volume was carried out in each vial prior to injection. As a recovery standard, 100 µL of PCB 209 at 617.28 µg L^−1^ was used. Calibrations were performed by using certified standard solutions of the analytes under study, which were injected in parallel with the sample at appropriate concentrations (multi-level calibration).

#### 3.4.2. GC-MS Analysis: Experimental Conditions A

The capillary column, RTx^®^-PCB Restek (Bellefonte, PA, USA) 60 m × 0.25 mm ID, 0.25 µm df, was connected to the mass spectrometer. Sample injections (2 µL) were performed in splitless mode: split off at 0.0 min, split ratio = 100 at 1.0 min. The injector temperature was 280 °C. The column oven temperature was programmed as follows: initial temperature 100 °C, 2.67 min hold; raising to 200 °C at a rate of 30 °C/min, 0 min hold; raising to 320 °C at a rate of 2 °C/min, 1 min hold. Helium, as the carrier gas, was used at a constant flow of 1.1 mL/min. Transfer line and ion trap temperatures were 280 and 240 °C, respectively. Full scan mass spectra were achieved through EI (electron ionization) in the acquisition range of 50–550 *m*/*z* and time range of 14–64 min.

#### 3.4.3. GC-MS Analysis: Experimental Conditions B

The capillary column, Chrompack^®^ CP SIL8 CB (Middelburg, The Netherlands) 30 m × 0.25 mm ID, 0.25 µm df, was connected to the mass spectrometer. Sample injections (2 µL) were performed in splitless mode: split off at 0.0 min, split ratio = 100 at 0.8 min. The injector temperature was 250 °C. The column oven temperature was programmed as follows: initial temperature 60 °C, 2.00 min hold; raising to 160 °C at a rate of 12.5 °C/min, 0 min hold; raising to 260 °C at a rate of 2 °C/min, 5 min hold. Helium, as the carrier gas, was used at a constant flow of 1.1 mL/min. Transfer line and ion trap temperatures were 250 and 210 °C, respectively. Full scan mass spectra were achieved through EI (electron ionization) in the acquisition range of 70–550 *m*/*z* and time range of 10–61 min.

## 4. Conclusions

The present work investigated the mass spectrometric ortho effect applied to the analysis of priority environmental contaminants. The ortho effect causes a high intensity of the first dehalogenation ion, which has previously been recognized as a potentially useful analytical tool. However, it was mainly studied on pure standards until now. In the present work, for the first time, the qualitative and quantitative reliability of the phenomenon was investigated on real samples of food by carrying out targeted experimental measurements using different detectors.

The good accuracy of the results obtainable by using the ortho effect was experimentally confirmed. In addition, it is shown here how to recognize the presence of a PCB isomer with no ortho effect when coeluting with an isomer that does. This is an important aspect because it relates to dioxin-like congeners and has never been studied until now.

The same ortho effect can also be seen in polybrominated biphenyls, as demonstrated by the mass spectra reported, and is also very likely in mixed poly-brominated/chlorinated biphenyls, for which few pure standards are still available, given that they are emerging contaminants.

## Figures and Tables

**Table 1:** Quantitative analysis of nine PCBs in Food Sample 1 (ng g^−1^). GC-MS: Sample injected with the same experimental conditions as in Section 3.4.2. GC-ECD: Sample injected with the same experimental conditions as in Section 3.4.1.

PCB	Intensity of Ortho Effect	Quantitative Analysis with GC-MS, [M^●+^]	Quantitative Analysis with GC-MS, [M-Cl]^+^	Quantitative Analysis with GC-ECD
49	39%	2.6	<LOQ	2.7
52	49%	7.8	7.6	8.5
87	41%	2.8	3.8	2.9
97	52%	2.1	2.8	2.0
132	48%	3.9	3.6	3.0
151	57%	4.1	4.2	4.3
153	20%	42.9	39.9	39.9
180	35%	19.5	18.0	19.0
187	47%	11.0	10.4	10.0

**Table 2:** Limit of detection (LOD) and limit of quantitation (LOQ) of the different detection methods used for the nine PCBs analyzed in Table 1 (ng g^−1^).

PCB	Quantitative Analysis with GC-MS, [M^●+^]		Quantitative Analysis with GC-MS, [M-Cl]^+^		Quantitative Analysis with GC-ECD	
	LOD	LOQ	LOD	LOQ	LOD	LOQ
49	0.7	1.0	3.0	4.0	0.09	0.14
52	0.4	0.6	1.9	2.9	0.14	0.21
87	0.6	0.8	1.1	1.6	0.08	0.12
97	1.4	2.1	1.9	2.8	0.09	0.14
132	0.8	1.2	2.4	3.6	0.09	0.14
151	0.5	0.8	2.1	3.2	0.09	0.13
153	1.1	1.6	7.3	10.9	0.16	0.23
180	1.4	2.2	4.0	6.0	0.13	0.19
187	0.9	1.4	2.3	3.5	0.11	0.17

## Data Availability

Data are contained within the article and Supplementary Materials.

## Supplementary Materials

The following supporting information can be downloaded at: https://www.mdpi.com/article/10.3390/molecules29153484/s1, Table S1: Intensity of the ortho effect measured in the present work for 37 PCBs; Figure S1: GC-MS chromatogram of five tetrachlorobiphenyls injected as a standard mixture at a concentration of 22.2 µg L^−1^; Table S2: Intensity of the ortho effect for 17 PBBs from the NIST 08 MS library.
