# Supplementary material for: The Mass Spectrometric Ortho Effect for Distinguishing the Coeluting Isomers of Polychlorinated Biphenyls and the Coeluting Isomers of Polybrominated Biphenyls: Qualitative and Quantitative Aspects
Source: Molecules. 2024 Jul 25;29(15):3484. doi: 10.3390/molecules29153484 (PMC11314496; doi:10.3390/molecules29153484)
Supplement: Supplementary file 1 [file molecules-29-03484-s001.zip › molecules-3112364-supplementary.pdf]

The mass spectrometric ortho effect for distinguishing the coeluting isomers of Polychlorinated Biphenyls and the coeluting isomers of Polybrominated Biphenyls: qualitative and quantitative aspects

Maurizio Masci [1,*]

[1] Council for agricultural research and economics (CREA), Research Centre for Food and Nutrition, via Ardeatina 546, 00178 Rome, Italy

* Correspondence: maurizio.masci@crea.gov.it

Table S1. Intensity of the ortho effect measured in the present work for 37 PCBs. Experimental conditions as in section 3.4.2. Comparison with the work of Osemwengie and Sovocool [20].

PCB	Type of Cl-substitution	Structural formula	Ortho effect measured in the present work (%)	Ortho effect measured by Osemwengie and Sovocool (%)
PCB 5	2,3 -	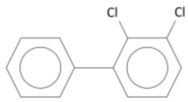	10	5
PCB 28	2,4,4′-	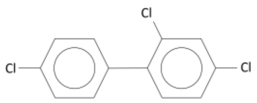	4	3
PCB 31	2,4′,5 -	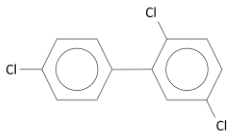	5	3
PCB 33	2′,3,4 -	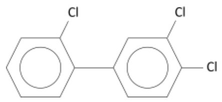	6	3
PCB 44	2,2′,3,5′ -	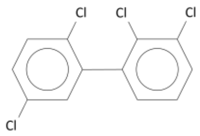	66	55
PCB 47	2,2′,4,4′-	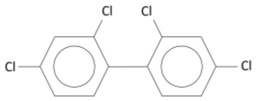	37	26
PCB 49	2,2′,4,5′-	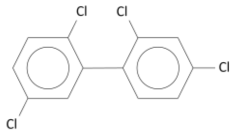	43	35

Table S1 (*continued*)

PCB	Type of Cl-substitution	Structural formula	Ortho effect measured in the present work	Ortho effect measured by Osemwengie and Sovocool (%)
PCB 52	2,2′,5,5′-	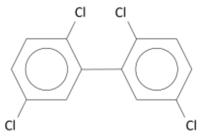	48	42
PCB 56	2,3,3′,4′-	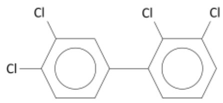	4	2
PCB 66	2,3′,4,4′-	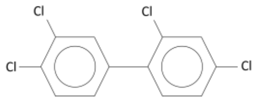	4	2
PCB 74	2,4,4′,5 -	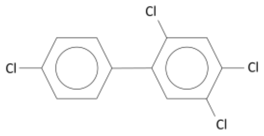	2	2
PCB 87	2,2′,3,4,5′-	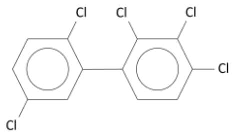	47	41
PCB 90	2,2′,3,4′,5 -	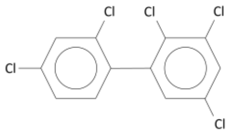	47	42
PCB 97	2,2′,3′,4,5 -	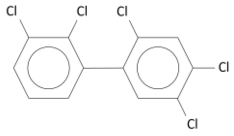	45	38
PCB 101	2,2′,4,5,5′-	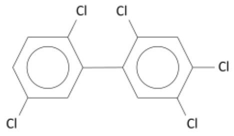	39	29
PCB 104	2,2′4,6,6′-	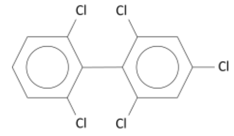	9	6
PCB 110	2,3,3′,4′,6 -	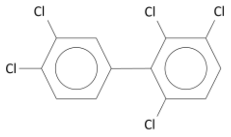	6	3
PCB 114	2,3,4,4′,5 -	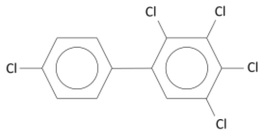	3	3

Table S1 (*continued*)

**PCB**	**Type of Cl-substitution**	**Structural formula**	**Ortho effect measured in the present work**	**Ortho effect measured by Osemwengie and Sovocool (%)**
PCB 118	2,3′,4,4′,5 -	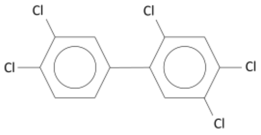	2	1
PCB 128	2,2′,3,3′,4,4′-	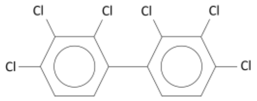	61	42
PCB 131	2,2′,3,3′,4,6 -	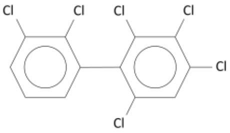	55	53
PCB 132	2,2′,3,3′,4,6′-	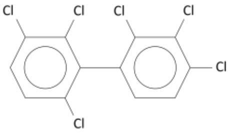	44	50
PCB 136	2,2′,3,3′,6,6′-	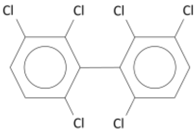	9	10
PCB 138	2,2′,3,4,4′,5′-	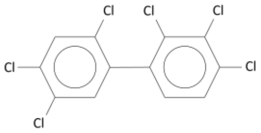	32	25
PCB 151	2,2′,3,5,5′,6 -	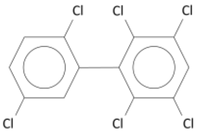	58	46
PCB 153	2,2′,4,4′,5,5′-	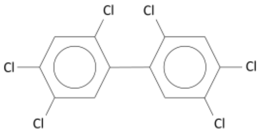	21	17
PCB 157	2,3,3′,4,4′,5′-	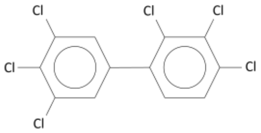	3	2
PCB 163	2,3,3′,4′,5,6 -	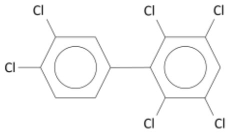	4	3
PCB 170	2,2′,3,3′,4,4′,5 -	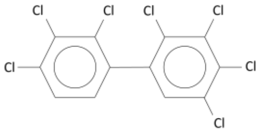	52	40

Table S1 (*continued*)

PCB	Type of Cl-substitution	Structural formula	Ortho effect measured in the present work	Ortho effect measured by Osemwengie and Sovocool (%)
PCB 178	2,2′,3,3′,5,5′,6 -	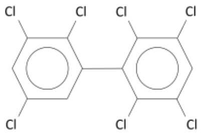	53	54
PCB 180	2,2′,3,4,4′,5,5′-	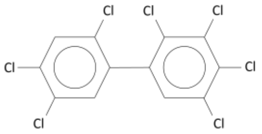	32	25
PCB 185	2,2′,3,4,5,5′,6 -	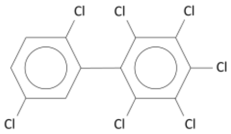	48	39
PCB 187	2,2′,3,4′,5,5′,6 -	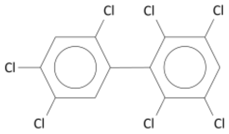	37	33
PCB 188	2,2′,3,4′,5,6,6′-	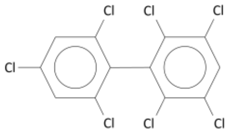	9	6
PCB 193	2,3,3′,4′,5,5′,6 -	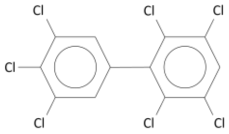	6	5
PCB 198	2,2′,3,3′,4,5,5′,6 -	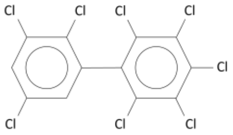	65	40
PCB 209	2,2′,3,3′,4,4′,5,5′,6,6′-	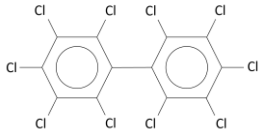	5	2

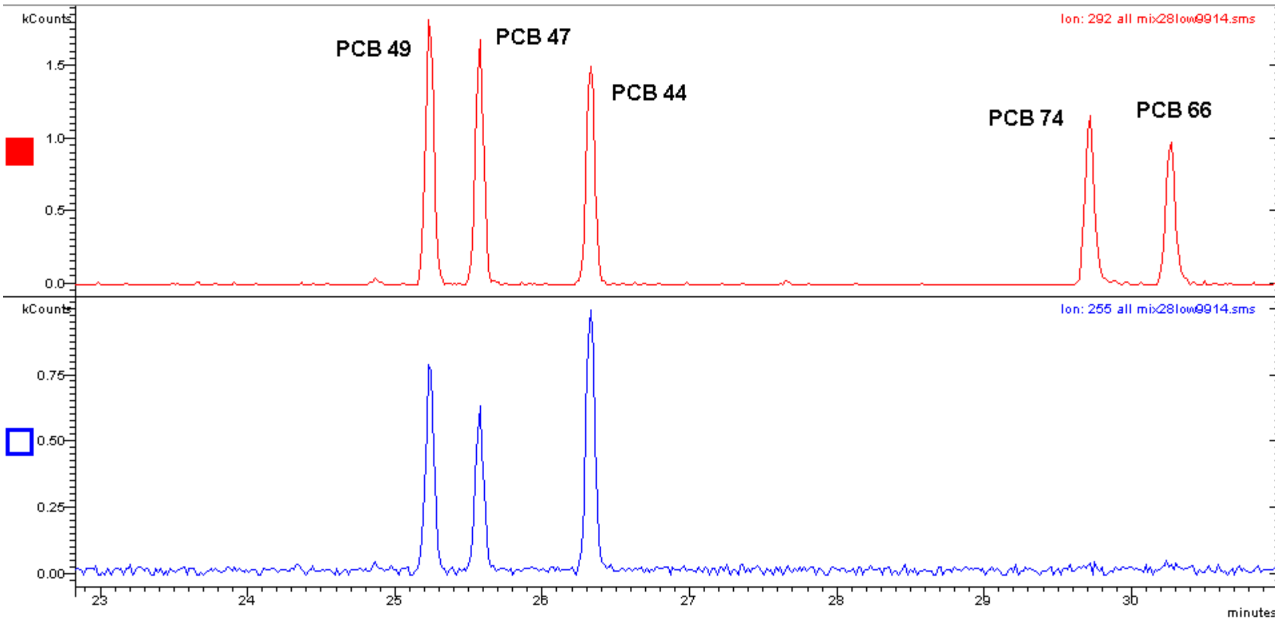

Figure S1. GC-MS chromatogram of five tetrachlorobiphenyls injected as a standard mixture all at a concentration of 22.2 μg L^{-1}. Top: chromatogram obtained by displaying the molecular ion 292 m/z. Bottom: chromatogram obtained by displaying the $[M\text{-}Cl]^+$ ion 255 m/z. Experimental conditions as in section 3.4.2.

In the chromatographic run of Figure S1 PCB 49, 47, and 44 have the following ortho effect, respectively: 43%, 37%, and 65%. PCB 74 and PCB 66 have both an ortho effect of 2%. It may be seen that, by recalling the $[M\text{-}Cl]^+$ ion 255 m/z, PCB 74 and 66 don't emerge from the baseline at all.

Table S2. Intensity of the ortho effect for 17 PBBs as from NIST 08 MS library.

PBB	Mass spectrum and structural formula	Ortho effect (%)
PBB 4	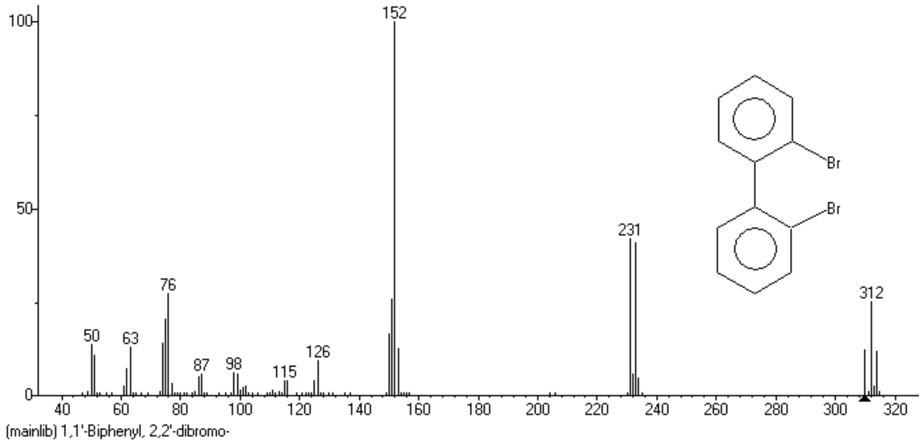	168
PBB 15	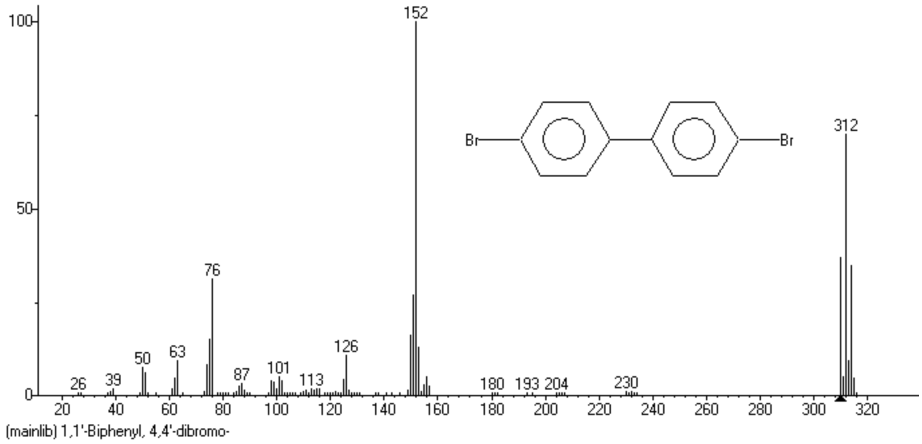	1
PBB 18	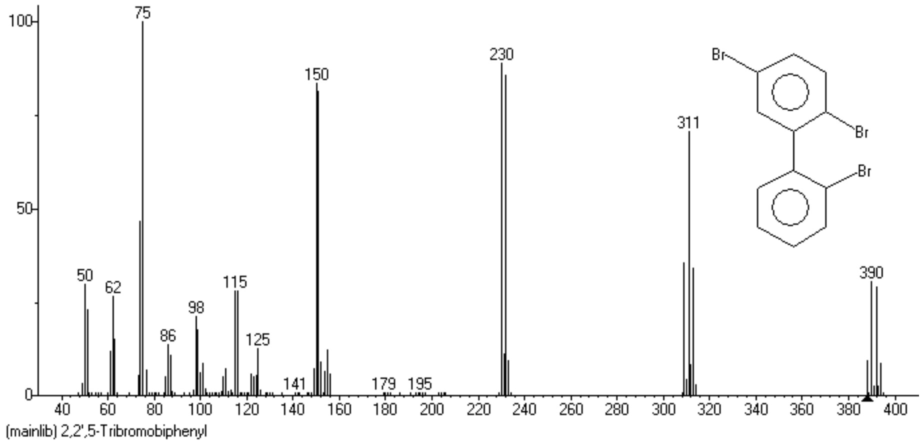	233

Table S2 *(continued)*

PBB	Mass spectrum and structural formula	Ortho effect (%)
PBB 26	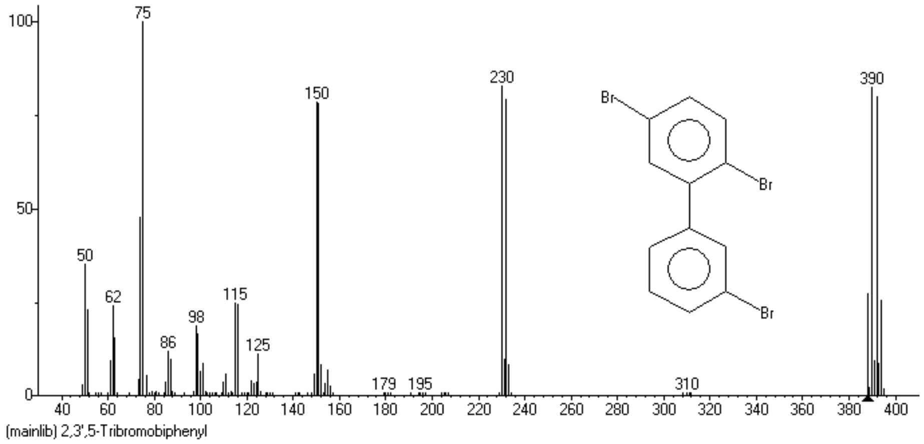	0.2
PBB 29	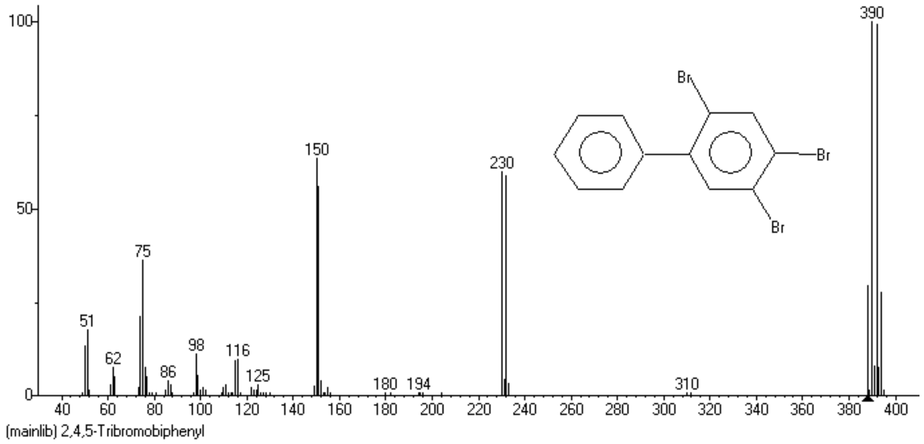	0.3
PBB 30	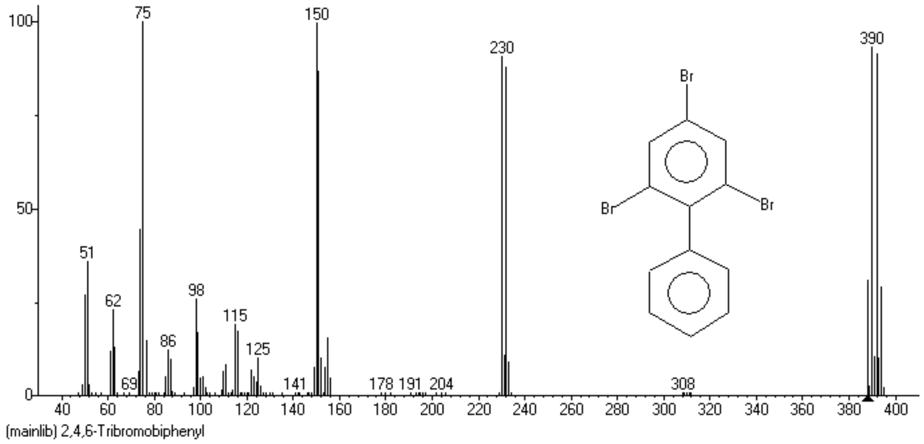	1

Table S2 *(continued)*

PBB	Mass spectrum and structural formula	Ortho effect (%)
PBB 31	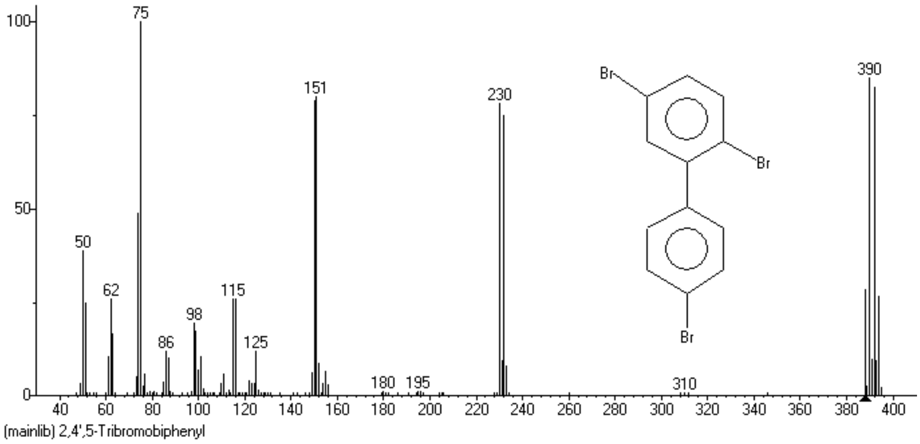	0.2
PBB 38	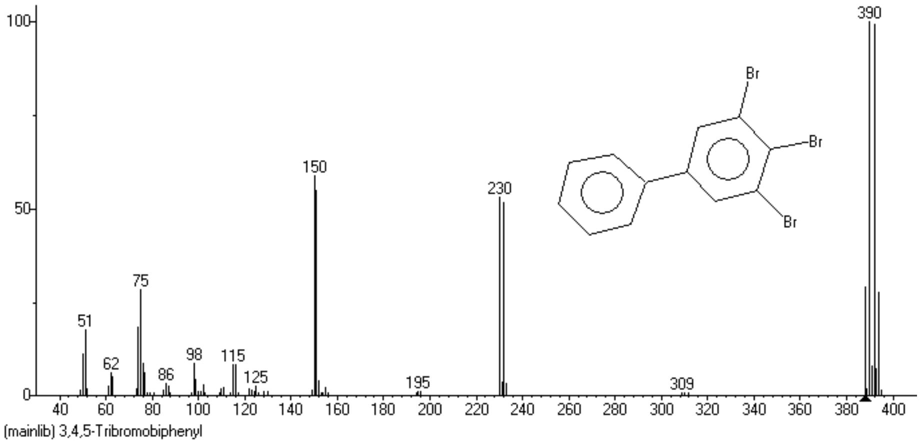	0.3
PBB 49	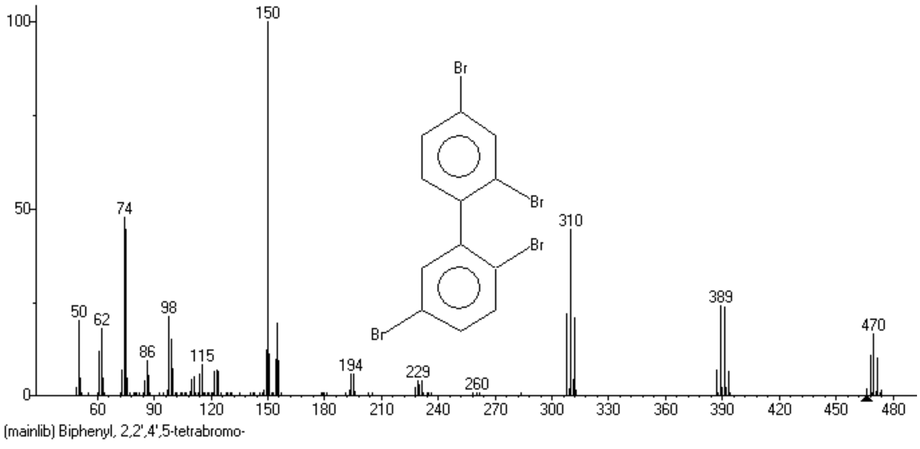	148

Table S2 *(continued)*

PBB	Mass spectrum and structural formula	Ortho effect (%)
PBB 52	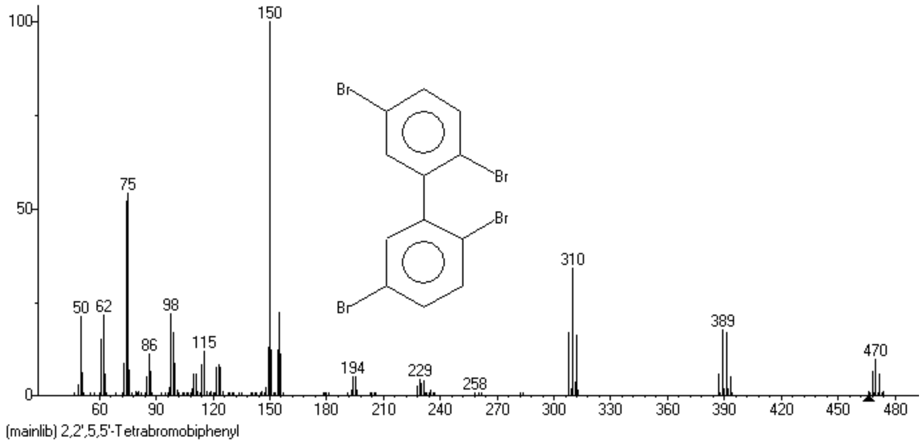	183
PBB 53	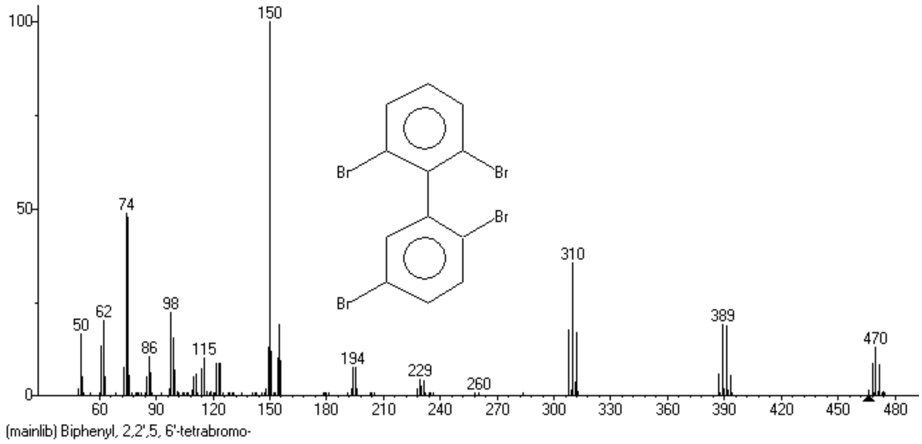	145
PBB 80	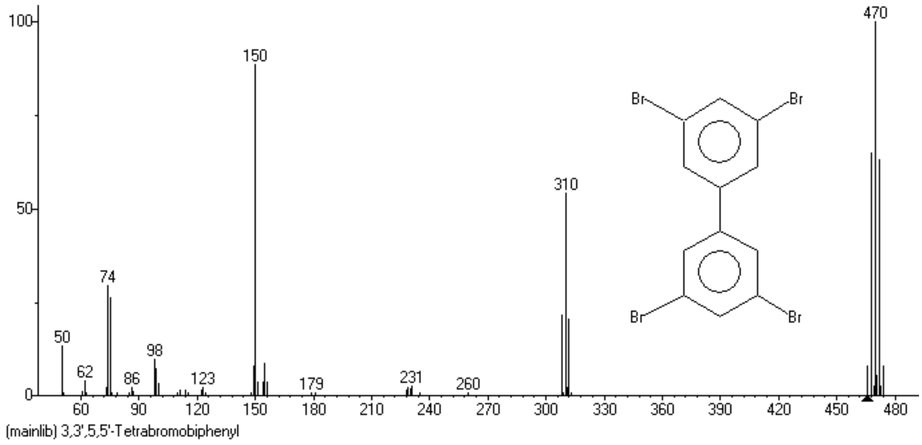	0

Table S2 *(continued)*

PBB	Mass spectrum and structural formula	Ortho effect (%)
PBB 101	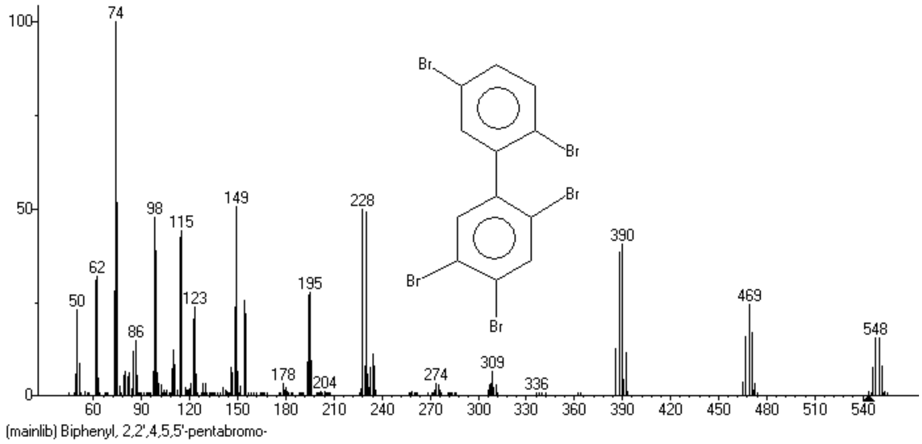	160
PBB 103	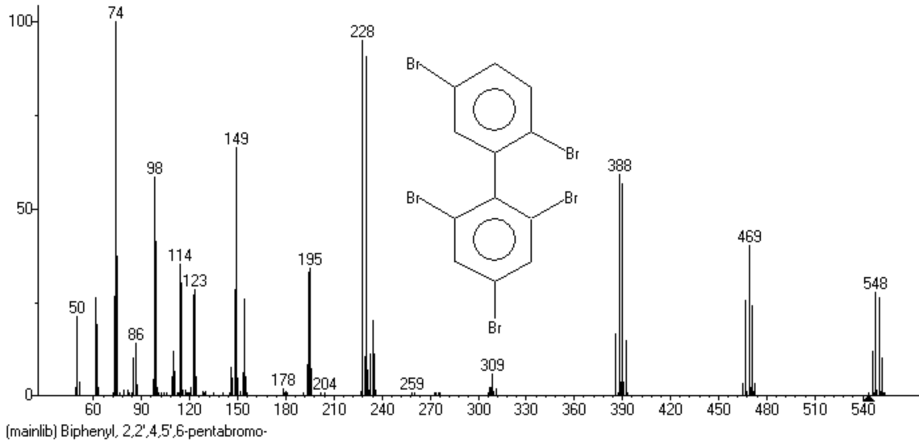	146
PBB 153	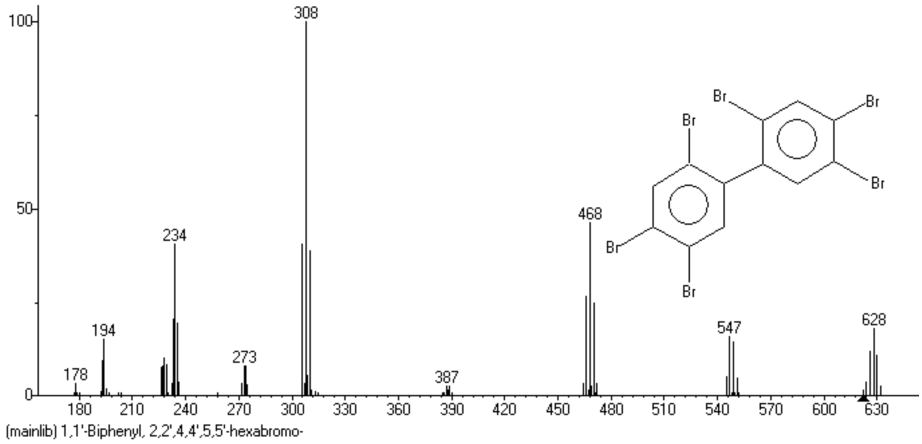	87

Table S2 *(continued)*

PBB	Mass spectrum and structural formula	Ortho effect (%)
PBB 155	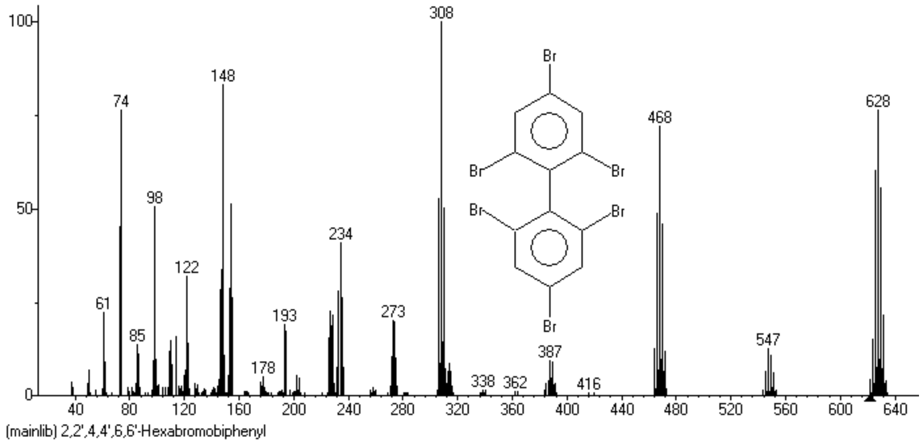	16
PBB 169	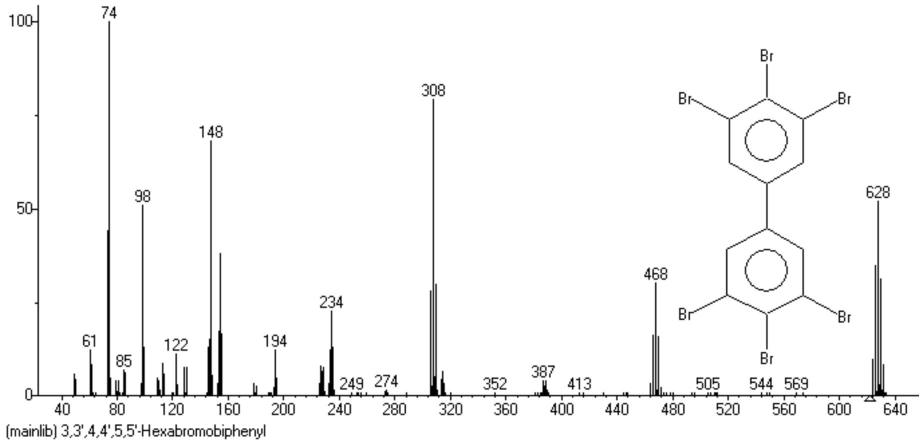	0.4
